# Anforderungen und Herausforderungen junger akademischer Chirurg*innen in der chirurgischen Onkologie

**DOI:** 10.1055/a-2739-3979

**Published:** 2025-12-01

**Authors:** Artur Rebelo, Andreas Brandl, Tobias Huber, Kim C. Honselmann, Rosa Klotz, Jörg Kleeff

**Affiliations:** 1 Universitätsklinik und Poliklinik für Viszerale, Gefäß- und Endokrine Chirurgie des Universitätsklinikums Halle (Saale) 9176 Martin-Luther-Universität Halle-WittenbergHalle (Saale)Germany; 2 Klinik für Allgemein-, Viszeral- und TransplantationschirurgieUniversitätsklinikum HeidelbergHeidelbergDeutschland; 3 Klinik für Allgemein-, Viszeral- und TransplantationschirurgieUniversitätsmedizin der Johannes Gutenberg-UniversitätMainzDeutschland; 4 Klinik für ChirurgieUniversitätsklinikum Schleswig-Holstein, Campus LübeckLübeckDeutschland

**Keywords:** Ausbildung, Onkologie, Chirurgie, training, oncology, surgery

## Abstract

**Hintergrund:**

Die akademisch-chirurgische Karriereentwicklung in Deutschland steht vor vielfältigen Herausforderungen. Junge Chirurg*innen sehen sich neben einer hohen klinischen Arbeitsbelastung auch mit einem Mangel an Fördermöglichkeiten und klaren Karrierepfaden konfrontiert. Gleichzeitig besteht ein ausgeprägtes Interesse an wissenschaftlicher Tätigkeit sowie an spezialisierten Ausbildungswegen wie Fellowships. Ziel dieser Arbeit war es, den aktuellen Stand der Weiterbildungsmöglichkeiten, die Interessen und die Bedürfnisse von Early-Career-Chirurg*innen im Bereich der chirurgischen Onkologie zu erheben, um gezielte Maßnahmen zur Förderung der wissenschaftlichen und klinischen Laufbahnentwicklung ableiten zu können.

**Material und Methoden:**

Ein Onlinefragebogen zu Karriereplanung, Forschungsinteressen, struktureller Unterstützung und Ausbildungserfordernissen wurde erstellt. Die Befragung richtete sich an chirurgisch tätige Ärzt*innen in Deutschland und wurde über chirurgische Fachgesellschaften sowie Netzwerke junger Chirurg*innen verbreitet. Die Ergebnisse wurden quantitativ ausgewertet.

**Ergebnisse:**

Es nahmen 191 Personen teil, die überwiegend in Universitätskliniken (45,1%) oder Häusern der Maximalversorgung (28,0%) tätig waren. Die größten Herausforderungen wurden in der klinischen Arbeitsbelastung (82,4%) und im Zeitmangel für Forschung (66,0%) gesehen. 85,4% zeigten Interesse an spezialisierten Fellowships, insbesondere in kolorektaler, hepatobiliärer oder Pankreaschirurgie. Der Zugang zu Mentor*innen (90,9%) und geschützte Forschungszeit (77,6%) wurde als essenziell erachtet. Forschungsinteresse bestand primär im Bereich klinischer (81,5%) und chirurgisch-technischer Forschung (62,3%). Die Einrichtung themenspezifischer Subgruppen innerhalb einer zukünftigen Early-Career-Group (ECG) – klinischen Studien, Robotik oder Mentoring – wurde von der Mehrheit der Teilnehmenden als hilfreich bewertet.

**Schlussfolgerung:**

Die Ergebnisse unterstreichen ein klares Interesse an wissenschaftlicher Tätigkeit und der Notwendigkeit strukturierter Förderung unter jungen Chirurg*innen. Gleichzeitig wird deutlich, dass bestehende Rahmenbedingungen, insbesondere im klinischen Alltag, einer aktiven wissenschaftlichen Beteiligung oft entgegenstehen. Die Diskrepanz zwischen hohem Interesse und begrenzten strukturellen Möglichkeiten verdeutlicht den Bedarf an gezielten Maßnahmen in Ausbildung, Mentoring und Forschungsförderung. Trotz hoher Motivation fehlt es jungen Chirurg*innen in Deutschland häufig an Unterstützung für eine wissenschaftlich-akademische Laufbahn. Die Einführung klarer Förderstrukturen, spezialisierter Fellowships und geschützter Forschungszeit erscheint essenziell, um die Zukunft der chirurgischen Onkologie nachhaltig zu sichern.

## Abstract (en)

**Purpose:**

Academic career development in surgical oncology in Germany faces multiple challenges. Young surgeons are confronted not only with high clinical workloads but also with a lack of structured support, funding opportunities, and transparent career pathways. At the same time, there is a strong interest in academic work and specialized training programs such as fellowships. The aim of this study was to assess the current state of training opportunities, interests, and needs of early-career surgeons in surgical oncology in order to derive targeted strategies to support their clinical and academic career development.

**Materials and Methods:**

An online survey covering career planning, research interests, structural support, and training needs was developed and distributed via surgical societies and networks for young surgeons across Germany. The results were analyzed quantitatively.

**Results:**

A total of 191 individuals participated, with most working at university hospitals (45.1%) or tertiary care centers (28.0%). The main challenges reported were high clinical workload (82.4%) and lack of time for research (66.0%). A strong interest in specialized fellowships was expressed by 85.4% of respondents, particularly in colorectal, hepatobiliary, or pancreatic surgery. Mentorship (90.9%) and protected research time (77.6%) were deemed essential. Research interests primarily focused on clinical (81.5%) and surgical-technical research (62.3%). The establishment of topic-specific subgroups within a future Early Career Group (ECG)—such as clinical trials, robotics, or mentoring—was considered helpful by the majority.

**Conclusion:**

The findings highlight a clear interest in academic engagement among young surgeons and the urgent need for structured support. However, current clinical working conditions often hinder active scientific involvement. The gap between motivation and structural limitations underlines the necessity for targeted measures in training, mentoring, and research support. Despite high motivation, young surgeons in Germany frequently lack sufficient support for pursuing an academic career.

## Hintergrund und Fragestellung

Die chirurgische Onkologie stellt hohe Anforderungen an junge Chirurg*innen in der Facharztausbildung sowie an junge Fach- und Oberärzt*innen. Neben der Beherrschung komplexer operativer Techniken müssen sie sich in einem zunehmend interdisziplinären und wissenschaftlich geprägten Umfeld behaupten. Gleichzeitig beeinflussen strukturelle Veränderungen im Gesundheitswesen, der zunehmende ökonomische Druck sowie neue Ausbildungs- und Karrieremodelle die berufliche Entwicklung dieser Zielgruppe. Um die spezifischen Herausforderungen und Bedürfnisse junger akademischer Chirurg*innen in der chirurgischen Onkologie besser zu verstehen, wurde eine Umfrage durchgeführt. Ziel dieser Studie war es, zentrale Problemfelder zu identifizieren und gezielte Unterstützungsmaßnahmen zu entwickeln, die die Karriereentwicklung, die Weiterbildungsmöglichkeiten und die wissenschaftliche Einbindung dieser Gruppe verbessern. Die Ergebnisse dieser Umfrage sollen dazu beitragen, bestehende Förderprogramme und Strukturen weiterzuentwickeln und an die Bedürfnisse junger Chirurg*innen in der onkologischen Viszeralchirurgie anzupassen.

Junge akademische Chirurg*innen stehen vor einem kontinuierlich größer werdenden Spagat zwischen klinischem Alltag und wissenschaftlicher Karriere. Strukturelle Fördermaßnahmen wie Mentoring, Forschungszeit und Karriereperspektiven sind bislang unzureichend etabliert.

Die zunehmende Ausrichtung vieler Krankenhäuser auf ein „For-Profit“-Modell beeinträchtigt die Förderung von Forschung und Lehre massiv. Hauptprobleme sind Zeit- und Ressourcenmangel für Forschung (79% der Nachwuchschirurg*innen), eine belastende Work-Life-Balance (64%) sowie unzureichende Mentoringprogramme (36% empfinden sie als oberflächlich). Zudem herrscht Intransparenz bei Führungs- und Auswahlprozessen, wobei wirtschaftliche Interessen scheinbar Vorrang haben. Eine Neuausrichtung der Führungskultur mit strukturierten Mentoringprogrammen, finanziellen Anreizen und festen Forschungszeiten könnte akademische Karrieren in der Viszeralchirurgie attraktiver machen 1 .

Führungsstrukturen, Diversität und psychosoziale Belastungen stellen zentrale Rahmenbedingungen dar, die die Karrierewege junger akademischer Chirurg*innen entscheidend beeinflussen. Trotz dieser Herausforderungen engagieren sich viele Chirurg*innen aktiv für bessere akademische Strukturen und transparente Führungsstrukturen. Die Analyse aktueller Literatur und Studien zeigt auch, dass der Führungsstil und die -struktur in der Chirurgie durch eine Kombination traditioneller Hierarchien und neuer Anforderungen geprägt sind. Der traditionelle hierarchische Führungsstil ist in chirurgischen Abteilungen weiterhin stark verankert, was sich in klaren Rollenverteilungen und Entscheidungswegen zeigt. Diese Struktur gewährleistet Stabilität, wird jedoch dafür kritisiert, dass sie Innovationen und die Einbindung jüngerer Kolleg*innen begrenzen kann. Ein weiterer kritischer Punkt ist die mangelnde Diversität in chirurgischen Führungspositionen. Unterrepräsentierte Gruppen haben es nach wie vor schwer, in leitende Positionen zu gelangen 2 3 4 5 6 7 . Ein weiterer kritischer Punkt ist der hohe Stresslevel, der sowohl bei Führungskräften als auch bei den Teams zu beobachten ist. Der Druck, klinische Exzellenz, Forschung und administrative Aufgaben zu vereinen, führt bei nicht wenigen Chirurg*innen zu Burn-out-Symptomen 8 9 . Ein Mangel an Führungsschulungen und Unterstützung während der chirurgischen Ausbildung kann dazu beitragen, dass junge Chirurg*innen schlecht auf die Anforderungen von Führungsrollen vorbereitet sind. Dieser Stress wird durch die unzureichende Implementierung von Unterstützungsprogrammen wie Mentoring oder Coaching weiter verstärkt, was langfristig die Resilienz und Zufriedenheit der Mitarbeitenden beeinträchtigt 3 . Die genannten Herausforderungen unterstreichen den dringenden Bedarf an strukturierten Förderprogrammen, die sowohl die wissenschaftliche als auch die klinische Entwicklung junger akademischer Chirurg*innen gezielt unterstützen. Eine zentrale Maßnahme ist die Einführung von geschützten Forschungszeiten, um wissenschaftliche Projekte ohne unmittelbaren klinischen Druck realisieren zu können 10 .

Während etablierte Mentoringprogramme in anderen Fachbereichen als Standard gelten, fehlen in der Chirurgie oft klare Strukturen für eine gezielte Karriereförderung. Junge Chirurg*innen profitieren besonders von fachspezifischen Netzwerken und strukturierten Karrierecoachings, die ihnen helfen, akademische und klinische Anforderungen besser zu vereinbaren. Die Etablierung solcher Programme kann zudem dazu beitragen, bestehende Hierarchien aufzubrechen und eine diversere Führungsebene in der Chirurgie zu fördern 11 .

Neben strukturellen Anpassungen ist auch eine kulturelle Veränderung innerhalb der chirurgischen Fachgesellschaften notwendig. Die Implementierung transparenter Karrierestrategien, gezielter Weiterbildungsmaßnahmen und finanzieller Anreize für wissenschaftliches Engagement könnte maßgeblich zur Attraktivität einer akademischen Laufbahn in der chirurgischen Onkologie beitragen.

In Deutschland vertritt die Arbeitsgemeinschaft „Junge Chirurgie“ (CAJC) die Interessen junger Chirurginnen und Chirurgen unter 40 Jahren und fördert ihre aktive Einbindung in die DGAV (Deutsche Gesellschaft für Allgemein- und Viszeralchirurgie). Im Fokus der CAJC stehen die Entwicklung von Strategien zur Nachwuchsgewinnung, die Definition optimaler Bedingungen für chirurgische Aus- und Weiterbildung sowie die Förderung von Karrierekonzepten insbesondere im Rahmen der Facharztweiterbildung. Ergänzend zur CAJC wird derzeit über die Gründung einer komplementären Early-Career-Gruppe innerhalb der DGAV-ACO (Assoziation Chirurgische Onkologie) nachgedacht. Diese soll gezielt Chirurg*innen nach abgeschlossener Facharztausbildung ansprechen, die sich auf eine Spezialisierung im Bereich der chirurgischen Onkologie vorbereiten oder bereits auf diesem Weg sind. Insbesondere in Deutschland fehlt bislang eine klar definierte Karrierebahn für viszeralchirurgisch tätige Ärzt*innen, die eine Spezialisierung in der chirurgischen Onkologie anstreben. Ob der Aufbau einer entsprechend strukturierten Fördergruppe notwendig und sinnvoll wäre, wurde bisher nicht systematisch untersucht. Eine solche Initiative könnte jedoch eine wichtige Lücke schließen und einen entscheidenden Beitrag zur Weiterentwicklung chirurgischer Karrieren leisten.

Ziel dieser Arbeit war es, die Herausforderungen, Interessen und Hürden der chirurgischen Nachwuchskräfte in der chirurgischen Onkologie systematisch zu erfassen.

## Studiendesign und Untersuchungsmethoden

### Studientyp

Bei dieser Untersuchung handelt es sich um eine deskriptive Querschnittsumfrage, die darauf abzielt, die Bedürfnisse und Herausforderungen junger akademischer Chirurg*innen in der chirurgischen Onkologie systematisch zu erfassen. Der eingesetzte, nicht validierte Fragebogen wurde im Rahmen eines interdisziplinären Konsensprozesses entwickelt. Diese methodische Einschränkung wurde bei der Interpretation der Ergebnisse berücksichtigt.

### Zielgruppe

Die Umfrage richtete sich an viszeralchirurgische onkologische Kolleg*innen:

1. Ärzt*innen in der Facharztausbildung
2. junge Fachärzt*innen
3. Oberärzt*innen ohne Leitungsposition oder Subspezialisierung

### Datenerhebung

Die Datenerhebung erfolgte über eine anonyme Onlineumfrage, die über das Umfragetool REDCap (Research Electronic Data Capture) bereitgestellt wurde. Die Umfrage wurde via E-Mail und andere Kommunikationsplattformen (z. B. LinkedIn) distribuiert. Die Datenerhebung fand im Februar und März 2025 statt.

### Fragebogen

Der Fragebogen umfasste 32 Fragen, die folgende Themenbereiche adressierten:

1. demografische Angaben (Alter, Geschlecht, Arbeitsort, berufliche Position)
2. Karrierepläne und Interessen (Spezialisierung, Motivation für die chirurgische Onkologie)
3. berufliche Herausforderungen (Zeitmangel, Karrieremöglichkeiten, Mentoring, Forschung)
4. Unterstützungsbedarfe (Mentoring, Weiterbildungsprogramme, Finanzierungsmöglichkeiten)
5. Work-Life-Balance und Vereinbarkeit von Forschung und klinischer Tätigkeit
6. Forschung und Weiterbildung (Interessen, geschützte Forschungszeit, Fellowships)
7. Subgruppenbildung innerhalb einer Early-Career-Gruppe

Die Teilnehmenden hatten die Möglichkeit, sowohl geschlossene als auch offene Fragen zu beantworten, um qualitative Aspekte einzubeziehen.

### Datenauswertung

Die gesammelten Daten wurden deskriptiv-statistisch analysiert, um Häufigkeiten, Mittelwerte und Verteilungen relevanter Merkmale darzustellen. Offene Antworten wurden inhaltsanalytisch kategorisiert. Alle eingegangenen Fragebogen wurden unabhängig vom Ausfüllgrad in die Auswertung einbezogen. Auch teilweise ausgefüllte Bogen wurden berücksichtigt, um möglichst alle verfügbaren Informationen zu nutzen.

### Ethik und Datenschutz

Die Teilnahme war freiwillig und anonym. Alle Daten wurden ausschließlich für wissenschaftliche Zwecke verwendet und in Übereinstimmung mit Datenschutzrichtlinien gespeichert und verarbeitet. Ein Ethikvotum wurde nicht eingeholt, da es sich um eine vollständig anonyme, freiwillige Onlineumfrage ohne Erhebung sensibler personenbezogener Daten handelte.

## Ergebnisse

### Demografische Daten der Teilnehmenden

Insgesamt nahmen 191 Chirurg*innen an der Umfrage teil. Von den eingegangenen Fragebogen wurden 73% vollständig ausgefüllt. Eine genaue Rücklaufquote lässt sich nicht angeben, da der Fragebogen u. a. über berufliche Netzwerke wie LinkedIn geteilt wurde und daher keine exakte Zahl der kontaktierten potenziellen Teilnehmenden vorliegt. Die Teilnehmenden waren überwiegend junge Chirurg*innen in der Weiterbildung oder frühe Fach- und Oberärzt*innen. Die Verteilung der aktuellen beruflichen Positionen war wie folgt: 52 (28,6%) waren Fachärzt*innen, 56 (30,8%) Oberärzt*innen, während 74 (40,7%) angaben, eine andere Position zu bekleiden, darunter Assistenzärzt*innen, Ärzt*innen in Weiterbildung, Funktionsoberärzt*innen u. a.

**Abb. 1:**
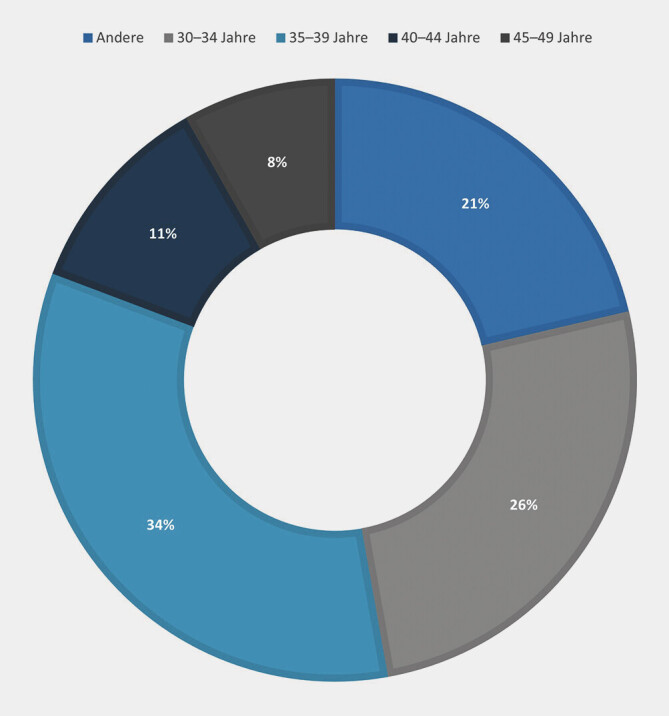
Altersverteilung. < 30 Jahre (13; 7,1%), 30–34 Jahre (47; 25,8%), 35–39 Jahre (61; 33,5%), 40–44 Jahre (20; 11,0%), 45–49 Jahre (15; 8,2%), 50–54 Jahre (10; 5,5%), 55–59 Jahre (11; 6,0%), > 60 Jahre (5; 2,7%).

Die Altersverteilung zeigt Abb. 1 .

Hinsichtlich des Geschlechts identifizierten sich 110 (60,4%) als männlich und 71 (39,0%) als weiblich.

Die Mehrheit der Befragten (82; 45,1%) war an Universitätskliniken tätig, gefolgt von Maximalversorgern (51; 28,0%) und Schwerpunktversorgern (38; 20,9%). Lediglich 11 (6,0%) arbeiteten in Krankenhäusern der Grund- und Regelversorgung.

### Karriereinteressen und Motivation

Ein bedeutender Teil der Befragten (111; 65,7%) plant eine Spezialisierung in der chirurgischen Onkologie, während 14 (8,3%) dies ausschlossen und 44 (26,0%) angaben, dies möglicherweise in Betracht zu ziehen.

Zu den Hauptmotiven für eine Karriere in der chirurgischen Onkologie zählten:

1. die Begeisterung für anspruchsvolle chirurgische Verfahren (142; 86,1%),
2. die Möglichkeit zur interdisziplinären Zusammenarbeit (121; 73,3%),
3. das Potenzial zur Verbesserung der Patientenversorgung (112; 67,9%),
4. die Möglichkeit zu Innovationen in der Behandlung (95; 57,6%) sowie
5. das Interesse an onkologischer Forschung (78; 47,3%).

Ein weiteres motivierendes Element für 61 (37,0%) war die Möglichkeit, eine Leitungsposition zu erreichen.

### Herausforderungen und Unterstützungsbedarf

**Abb. 2:**
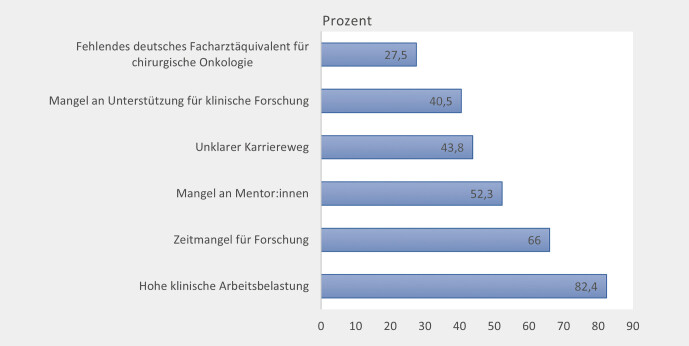
Herausforderungen in der Karriereentwicklung.

Die größten Herausforderungen in der Karriereentwicklung wurden von den Befragten in erster Linie in der hohen klinischen Arbeitsbelastung gesehen, die von 126 (82,4%) als problematisch eingestuft wurde. An 2. Stelle stand der Zeitmangel für Forschung (101; 66,0%), gefolgt von einem Mangel an Mentor*innen (80; 52,3%) und einem unklaren Karriereweg (67; 43,8%). Darüber hinaus gaben 62 (40,5%) der Befragten an, dass es an Unterstützung für klinische Forschung mangele. Ein weiteres Hindernis sahen 42 (27,5%) im fehlenden deutschen Facharztäquivalent für die chirurgische Onkologie. Finanzielle Unsicherheit wurde von 18 (11,8%) als Problem benannt, während 31 (20,3%) eine unklare Dauer ihres Arbeitsverhältnisses als herausfordernd empfanden (siehe Abb. 2 ).

Die Mehrheit der Teilnehmenden bewertete den Zugang zu strukturierten Unterstützungsprogrammen als essenziell:

1. 139 (90,9%) hielten den Zugang zu Mentor*innen für wichtig oder sehr wichtig.
2. 104 (77,6%) bewerteten geschützte Zeit für Forschung und Fortbildung als wichtig oder sehr wichtig.
3. 130 (87,0%) forderten eine stärkere Unterstützung durch Klinikleitung und Administration.

### Forschung und Weiterbildung

Ein Großteil der Befragten zeigte ausgeprägtes Interesse an wissenschaftlicher Arbeit:

1. 119 (81,5%) waren an klinischer Forschung interessiert,
2. 91 (62,3%) zeigten Interesse an chirurgisch-technischer Forschung,
3. 37 (25,3%) befürworteten translationale Forschung und
4. 22 (15,1%) interessierten sich für Grundlagenforschung.

Hinsichtlich der akademischen Karriere hielten 109 (75,2%) eine wissenschaftliche Laufbahn für hoch oder sehr hoch relevant. Zudem gaben 113 (85,4%) an, dass finanzielle Unterstützung für Forschungstätigkeiten essenziell sei.

Für eine gezielte Förderung wünschen sich die Teilnehmenden:

1. strukturierten Zugang zu Forschungsgruppen für Early-Career-Chirurg*innen (70; 50%),
2. finanzielle Unterstützung für Forschungsprojekte (86; 62,1%) und
3. spezialisierte Weiterbildungsprogramme (123; 85,4%).

### Work-Life-Balance und Karriereplanung

Eine gute Work-Life-Balance wurde von 97 (64,7%) der Befragten als wichtig oder sehr wichtig bewertet. Gleichzeitig äußerten 121 (82,3%), dass sie von einer strukturierten Karriereplanung profitieren würden.

Für eine bessere Karriereförderung wünschen sich 99 (69,7%) gezielte Workshops zu Karrierewegen in der chirurgischen Onkologie, ergänzt durch Themen wie Zeitmanagement, Netzwerkbildung und Finanzierungsmöglichkeiten.

### Fellowships und Subgruppen für Early-Career-Chirurg*innen

Das Interesse an Fellowships innerhalb Deutschlands war hoch: 123 (85,4%) der Teilnehmenden äußerten Interesse an einer spezialisierten Fellowship. Bevorzugte Subspezialisierungen waren:

1. kolorektale Chirurgie (75; 52,8%)
2. hepatobiliäre Chirurgie (72; 50,7%)
3. Pankreaschirurgie (69; 48,6%)
4. oberer GI-Trakt (62; 43,7%)
5. peritoneale Karzinose und HIPEC (40; 28,2%)
6. Sarkomchirurgie (20; 14,1%)

Zur Förderung des wissenschaftlichen Austauschs wurde eine thematische Subgruppenbildung für Early-Career-Chirurg*innen als nützlich bewertet. 97 (67,3%) hielten die Etablierung solcher Gruppen für hilfreich oder sehr hilfreich, und 74 (51,7%) erklärten sich bereit, aktiv mitzuwirken.

Die bevorzugten Themen für Subgruppen waren:

1. klinische Studien und Register (93; 66,9%)
2. onkologische Roboterchirurgie (74; 53,2%)
3. Karriereentwicklung und Mentoring (74; 53,2%)
4. Balance zwischen Klinik und Forschung (63; 45,3%)
5. Finanzierungs- und Stipendienberatung (36; 25,9%)

## Diskussion

Die vorliegenden Ergebnisse zeigen, dass junge Chirurg*innen ein hohes Maß an Interesse an wissenschaftlicher Arbeit und strukturierten Fördermaßnahmen aufweisen. Insbesondere klinische Forschung, Mentoring sowie gezielte Weiterbildungsprogramme werden als zentrale Bausteine einer erfolgreichen Karriereentwicklung benannt. Gleichzeitig verdeutlichen die Daten die erheblichen strukturellen Herausforderungen im klinischen Alltag, allen voran die hohe Arbeitsbelastung und der Zeitmangel für Forschung. Das starke Interesse an spezialisierten Fellowships sowie die Präferenz bestimmter chirurgischer Subspezialisierungen spiegeln ein wachsendes Bedürfnis nach vertiefter fachlicher Expertise innerhalb der chirurgischen Onkologie wider.

Diese Ergebnisse stehen im Einklang mit früheren Analysen der akademischen Chirurgie in Deutschland 12 . Insbesondere die hohe klinische Arbeitsbelastung (82,4%) und der Zeitmangel für Forschung (66,0%) stellen zentrale Hindernisse dar. Diese Beobachtungen decken sich mit früheren Untersuchungen, die zeigen, dass klinische Verpflichtungen und fehlende geschützte Forschungszeiten maßgeblich zur geringen wissenschaftlichen Aktivität junger Ärzt*innen beitragen 1 7 . Die Einführung strukturierter Clinician-Scientist-Programme mit garantierten Forschungszeiten zeigen jetzt schon hohe Potenziale zur Förderung akademischer Karrieren und sollten insbesondere für Chirurgen ausgerichtet werden 8 . Auch die Entwicklung eines Kooperationsnetzwerks innerhalb der ACO in Form eines Early-Career-Group-Programms, das sich an junge akademische Chirurg*innen nach der Facharztausbildung richtet, könnte eine gezielte Maßnahme sein, um diese Hürden zu überwinden. Im Vergleich zu anderen Ländern ist die strukturierte wissenschaftliche Zusammenarbeit in Deutschland noch wenig entwickelt, sodass die Vernetzung von Chirurg*innen in frühen Karrierephasen von entscheidender Bedeutung sein kann. Zudem könnte eine gezielte Unterstützung bei der Karriereentwicklung hin zum Fellow of the European Board (FEBS) of Surgical Oncology in Verbindung mit der Absolvierung des ACO-Curriculums eine sinnvolle und nachhaltige Maßnahme darstellen 13 .

Darüber hinaus unterstreichen die Studienergebnisse die Bedeutung von Mentoringprogrammen, da 90,9% der Teilnehmenden den Zugang zu Mentor*innen als wichtig oder sehr wichtig einstuften. Gleichzeitig gaben jedoch viele Befragte an, dass ihnen eine systematische Mentorenbetreuung fehlt. Ähnliche Defizite wurden bereits in anderen chirurgischen Fachbereichen identifiziert, in denen Mentoring oft informell und unstrukturiert erfolgt, was den Zugang zu akademischen Karrieren erschwert 2 13 . Studien zeigen, dass gezielte Mentoringstrukturen nicht nur die wissenschaftliche Produktivität erhöhen, sondern auch die Zufriedenheit und langfristige Karrieremotivation stärken 14 . Besonders für unterrepräsentierte Gruppen könnten strukturierte Programme eine wesentliche Maßnahme sein, um Barrieren auf dem Weg zu Führungspositionen abzubauen 3 8 . Dies fällt am ehesten in den Aufgabenbereich chirurgischer Fachgesellschaften, wie bspw. der Deutschen Gesellschaft für Allgemein- und Viszeralchirurgie (DGAV), da Mentorenprogramme häufig von Instituten allein nicht getragen werden können bzw. ein institutsfremder Mentor den Blickwinkel erweitern kann.

Ein weiteres zentrales Ergebnis dieser Studie ist das starke Interesse an spezialisierten Weiterbildungsprogrammen, insbesondere in den Bereichen kolorektale Chirurgie, hepatobiliäre Chirurgie, Pankreaschirurgie und oberer GI-Trakt. Dies spiegelt den wachsenden Bedarf an vertiefter operativer Expertise innerhalb der chirurgischen Onkologie wider. Ähnliche Entwicklungen wurden in internationalen Studien beobachtet, die zeigen, dass spezialisierte Fellowships nicht nur die chirurgische Weiterbildung verbessern, sondern auch die wissenschaftliche Produktivität steigern können 4 14 . Eine gezielte Förderung solcher Programme könnte daher einen wichtigen Beitrag zur akademischen Weiterentwicklung junger Chirurg*innen leisten.

Wie bereits erwähnt, zeigt die Umfrage, dass junge Chirurg*innen in der onkologischen Chirurgie zunehmend mit der Herausforderung der tiefgreifenden Spezialisierung konfrontiert sind. Während früher ein „allgemeiner Chirurg“ viele Bereiche abdeckte, erfordert die heutige komplexe onkologische Chirurgie zunehmend eine fokussierte Ausbildung. Internationale Modelle, wie sie z. B. in den Niederlanden praktiziert werden, zeigen, dass eine gezielte Spezialisierung notwendig ist, um eine hohe Qualität der Versorgung zu gewährleisten. Dennoch führt diese Entwicklung zu einer geringeren Zahl an Spezialist*innen, was sowohl Vorteile als auch Herausforderungen mit sich bringt. Es ist daher erforderlich, auch die Ausbildungskonzepte hierzulande zu überdenken und grundlegend anzupassen.

Ein weiterer wesentlicher Punkt ist die Rolle der Mentor*innen. Um eine qualitativ hochwertige Ausbildung zu gewährleisten, müssen Mentor*innen genügend Zeit für die Betreuung ihrer Mentees haben. Diese „freie Zeit“ sollte außerhalb der regulären klinischen Verpflichtungen, aber innerhalb der regulären Arbeitszeit möglich werden, um die Bedürfnisse beider Seiten zu berücksichtigen. Regelmäßige, strukturierte Debriefings und Fallbesprechungen wären ideal, stoßen jedoch aufgrund von Personalmangel häufig an ihre Grenzen. Ohne die nötigen Ressourcen und strukturellen Anpassungen wird es herausfordernd sein, die Anforderungen der Nachwuchschirurg*innen effektiv umzusetzen.

Diese Erhebung unterliegt mehreren Limitationen. Zum einen handelt es sich um eine freiwillige, anonyme Befragung, die über mehrere Kanäle verteilt wurde und somit eine mögliche Selektionsverzerrung beinhaltet – insbesondere durch eine potenzielle Überrepräsentation forschungsaffiner Teilnehmender. Zudem richtete sich die Erhebung ausschließlich an viszeralchirurgisch-onkologische Kolleg*innen, wodurch potenziell relevante Perspektiven aus angrenzenden chirurgischen oder onkologischen Fachbereichen nicht erfasst wurden. Zum anderen wurden keine validierten Fragebogen eingesetzt. Die Interpretation der Ergebnisse kann zudem durch die unterschiedliche Größe und Verteilung der Arbeitsorte beeinflusst sein. Es kann auch nicht ausgeschlossen werden, dass auch Personen außerhalb der ursprünglich definierten an der Umfrage teilgenommen haben. Diese potenzielle Heterogenität der Teilnehmenden könnte die Ergebnisse beeinflusst haben.

Ein wesentlicher Vorteil dieser Untersuchung liegt in der breiten Beteiligung chirurgisch tätiger Ärzt*innen verschiedener Versorgungsstufen, von der Universitätsklinik bis zur Grund- und Regelversorgung. Dadurch entsteht ein differenziertes Bild der aktuellen Herausforderungen und Bedürfnisse von Early-Career-Chirurg*innen in Deutschland. Die Erhebung ermöglicht damit eine praxisnahe Ableitung von Maßnahmen zur Verbesserung der Nachwuchsförderung und akademischen Entwicklung in der chirurgischen Onkologie.

Die Notwendigkeit einer Early-Career-Gruppe der ACO wird durch die Ergebnisse der aktuellen Umfrage klar unterstrichen: Junge akademische Chirurg*innen in der chirurgischen Onkologie sehen sich einer hohen klinischen Arbeitsbelastung bei gleichzeitig begrenzten Ressourcen für Forschung gegenüber. Trotz eines ausgeprägten Interesses an wissenschaftlicher Tätigkeit bestehen deutliche strukturelle Defizite hinsichtlich Karriereplanung sowie operativer und akademischer Förderung. Mentoring, geschützte Forschungszeiten sowie finanzielle und infrastrukturelle Unterstützung werden von den Befragten als zentrale Elemente einer erfolgreichen beruflichen Entwicklung identifiziert.

Diese Erkenntnisse machen deutlich, dass gezielte Förderprogramme, klar strukturierte Karrierepfade sowie subdisziplinäre Netzwerke für onkologisch tätige junge Chirurg*innen erforderlich sind. Die geplante Early-Career-Gruppe ACO soll diese Lücke schließen und somit einen entscheidenden Beitrag zur nachhaltigen Förderung des chirurgischen Nachwuchses im Bereich der Onkologie leisten.

## Fazit für die Praxis

Die Umfrage zeigt: Junge akademische Chirurg*innen in der onkologischen Chirurgie stehen unter hoher klinischer Belastung bei gleichzeitig eingeschränkten Forschungskapazitäten. Trotz großen Interesses an wissenschaftlicher Arbeit fehlen strukturierte Karrierewege und gezielte Förderangebote. Mentoring, geschützte Forschungszeit und finanzielle Unterstützung werden als entscheidend für die berufliche Entwicklung gesehen. Die Ergebnisse belegen den klaren Bedarf an spezifischen Programmen und Netzwerken – wie der geplanten Early-Career-Gruppe ACO.
